# Schooltime's contribution to pupils' physical activity levels: A longitudinal study

**DOI:** 10.3389/fpubh.2023.1100984

**Published:** 2023-02-06

**Authors:** Martine Amalie Johansen, Hilde Kristin Mikalsen, Pål Arild Lagestad

**Affiliations:** Department of Teacher Education and Art, Nord University, Levanger, Norway

**Keywords:** children, school time, physical activity level, health recommendations, longitudinal

## Abstract

**Introduction:**

Pupils spend a significant portion of their time at school. As a result, the school serves as an important setting for both learning and the formation of healthy behaviors. Many children, and even fewer young people, do not fulfill the (inter)national health recommendations of 60 minutes of moderate to vigorous physical activity (MVPA) per day. The aim of this study was to examine pupils' MVPA during schooltime in a longitudinal perspective, including the transition from primary to secondary school.

**Methods:**

The MVPA of 234 pupils' was measured objectively using accelerometer monitors for seven consecutive days, in the spring of 2017, 2018, and 2019. Statistical analyses by Friedman, Wilcoxon and Mann–Whitney *U*-test were used to answer the research questions.

**Results:**

The results showed a significant decrease in the pupils' MVPA and fulfilment of health recommendations during schooltime, from 7^th^ to 8^th^ grade. The analyses also showed that MVPA during schooltime was higher among boys than girls, and also contributed more to boys' fulfilment of the health recommendations at all three time periods.

**Discussion:**

The results indicate that the transition between primary and secondary school is vulnerable concerning pupils' MVPA during schooltime. As schooltime accounted for significantly more MVPA for boys than for girls at all three time periods, we question whether physical activity is sufficiently facilitated for girls in school.

## Introduction

National and international recommendations recommend that children and adolescents should be in moderate or vigorous physical activity (MVPA) for a minimum of 60 min daily (1, 2). The Directorate of Health (2) emphasizes the importance of regular physical activity (PA) as necessary for children and young people's normal growth and development. Physical inactivity, on the other hand, is associated with a variety of risk factors such as cardiometabolic disease, type-2 diabetes, cancer, and obesity (2–4). Physical inactivity also seems to have a negative impact on academic achievement and mental health (5, 6).

Bergqvist-Norén et al. (7) found that accelerometer-measured PA increased similarly for boys and girls by 11% per year from 2 years of age onwards to 6 years of age. However, studies show that the level of PA among young people reduces with age, particularly in the transition from childhood to adolescence (8–10). A cross-sectional study reports that the total amount of PA decreased by an average of 4.2% each year from the age of 5 to 18 (11). The proportion of 15-year-old girls and boys who attain the recommended levels of PA in Norway is 40 and 51% respectively (12). For this reason, we will argue that school can serve as an important setting for young people's PA, as children and adolescents spend a significant portion of their day there, and because all young people are included (6, 13). Good habits of PA in childhood can further contribute to the establishment of a pattern of activity that is carried forward into adulthood (14, 15). However, Díaz et al. (16) found that preschool children do not move enough, and that the school contributes little to the PA levels recommended to obtain health and the quality of life of pre-school children. More knowledge is needed about the contribution of PA in schooltime in the transition from childhood to adolescence.

Physical education (PE) has been pointed to as a subject able to raise the level of the pupils' MVPA (10, 17–19). The results from Andersen (19) show that PE accounted for around 4% of the weekly fulfillment of recommended MVPA. The same study showed that activity during breaks in the school day represented 18.2 and 6.8% of the recommended weekly total of boys and girls, respectively. Further, Andersen (19) found that 9-year-old pupils use 27% of their break time in MVPA, while 15-year-olds use only 10.5% of theirs in MVPA, and that, therefore, break time is especially important for pupils' MVPA in primary school, but less so in secondary school. There have been, however, few studies into pupils' MVPA in Norwegian schools. These are mainly cross-sectional studies; no longitudinal studies are looking at changes in children's and adolescents' PA during schooltime over time. Two published, Norwegian studies have looked at pupils' MVPA in schooltime (19, 20). Andersen (19) found that 9-year-olds were in MVPA for 28 min daily during schooltime, whilst the total for 15-year-olds was 19 min. Calculations based on the numbers from this study show that 6-, 9- and 15-year-olds reached 39, 34 and 23% respectively of the weekly target during school hours. Similarly, Harding et al. (21) found a reduction of 2.5 min from when pupils were 12-years-old to them being 15-years-old. Kristiansen et al. (20) found, in their study, that the time the pupils spent at school accounted for 30.6 and 26% of boys' and girls' total MVPA respectively. Both Andersen (19) and Kristiansen et al. (20) found boys' MVPA in schooltime to be significantly higher than girls'. In relation to the recommended healthy level, schooltime accounted for 45.9% (boys) and 36.6% (girls) of the time in MVPA required to reach that weekly level (20). Harding et al. (21) found that schooltime accounted for 31 and 28%, respectively, of 12- and 15-year-olds' attainment of 60 min weekly MVPA.

In Norway, most pupils change school when they start secondary school (22). For many, this transition brings a raft of changes, on both environmental and personal levels. Environmentally changes imply altered school settings with the new school location, new teachers, subjects, and class-mates. Personal changes concerning maturing and puberty occur during this transitional period. According to previous research (6, 23, 24) such transitions, which include multiple changes, make people more vulnerable in relation to coping and understanding their everyday life.

Remmers et al. (25) studied changes in PA among pupils in the Netherlands during this transition. All pupils in their study had to change school. They found that MVPA in schooltime went down by 12.5%. Similarly, Lau et al. (26) found that pupils' MVPA, both boys and girls, during schooltime went down between years 5 and 7. However, research also suggests that, compared to primary school, secondary school involves more responsibility, greater freedom and more choice (24, 27). Greater freedom and more self-determination has been shown to increase pupils' level of activity in PE classes (28). The presented research supports an ecological theory perspective on PA behaviour, implying that human behavior and development are dynamic products of interactive relations between all variables in the individual and all variables in the individual's context (29). We consider the ecological theory perspective, which is applied within developmental psychology, appropriate for our work of research.

Several studies have established that school's contribution to MVPA is significantly higher for boys, than for girls (19, 20, 25, 26). However, how PA and these gender differences are changed among the same pupils during the transition to secondary school, has not been studied. Based on this discussion, this study has three research questions, each of which gender differences will be highlighted:

1. To what extent does pupils' weekly MVPA during schooltime change between years 7, 8, and 9?
2. To what extent does schooltime's contribution to pupils' weekly MVPA change between years 7, 8, and 9, where pupils' attainment of total weekly MVPA is concerned?
3. To what extent does schooltime's contribution to pupils' weekly MVPA change between years 7, 8, and 9, where attainment of the health authorities' recommended MVPA is concerned?

## Method

The research questions are examined by using a longitudinal design with accelerometer measurements of PA levels among the same pupils during 3 years. The study has been approved by the Norwegian Centre for Research Data (NSD). Both parents and pupils gave informed written consent to participation. The study is in accordance with the declaration of Helsinki.

### Sample

Two medium-sized municipalities in the mid-Norway were chosen with the help of a stratified selection. In all, 18 of the total of 19 primary schools in these municipalities participated in the study (one of the smaller schools declined to participate). Pupils from the 18 primary schools were subsequently spread between 4 different secondary schools in the same municipalities in 2018 and 2019. At the start of the data collection in 2017, the sample consisted of 320 12–13-year-old pupils (7^th^ grade), who each had valid measurements of their activity from all three measurement periods, representing a 77% response rate. In all, 234 (124 girls and 110 boys) had valid measurements for all three time periods, concerning answering research questions 1 and 2, a response rate of 56%. Only 91 pupils (61 girls and 36 boys) had valid data for all three time periods regarding research question 3 – a 23% response rate. A sample size calculator show that we needed more than 30 participants to fulfil the criteria for observed power, and our study includes a much higher number of participants.

### Procedures

In this study, PA is defined as MVPA, which is the gold standard for the measurement of overall PA (30, 31), and in line with both national (2) and international (1) recommendations. All measurements were taken during the same week in April 2017, 2018, and 2019. MVPA was measured with the use of accelerometer ActiGraph GT1M, which is an approved and reliable instrument for the measurement of PA (32, 33). The pupils were instructed to carry the accelerometer on their right hip for 7 days, except only during water activities as the monitors do not tolerate water (30), and at night when sleeping. In keeping with large Norwegian population studies, the criterion for valid measurements was set at data over 8 h daily for at least 2 days, and MVPA was characterized as >2000 counts per min (12, 30). The storage interval for the raw data was set at 10 s. All data collection, from all measurements, was done by the same test leader. The same equipment was used throughout, and the procedures used were constant.

Data from the data collection was downloaded to Actilife v6.13.3 (ActiGraph, LLC, Pensacola, FL), where they were filtered and MVPA during schooltime separated from MVPA in leisure time. For the pupils' data to be valid, they must have a total of 480 min of counts (12). Periods of more than 20 min with no counts were not included, nor was activity between 00.00 and 06.00. The pupils must have a minimum of 180 min of counts for the school day to be considered valid.

### Analyses

The data material was analyzed using the program SPSS, version 27 (SPSS Inc., Chicago, IL, USA). As most of the dependent variables were not normally distributed according to the Kolmogorov Smirnov test (*p* < 0.05), non-parametric tests were used (34). Friedmann's test was used to study differences in MVPA during schooltime, and schooltime's contribution to the health recommendations over the three time periods, with the Wilcoxon test as a follow-up test to find pair-wise differences between the different years, using Bonferroni corrections. Finally, the Mann–Whitney *U*-test was used to examine gender differences. The significance level was set at *p* < 0.05, *p* < 0.01, and *p* < 0.001 respectively.

## Results

**Figure 1:**
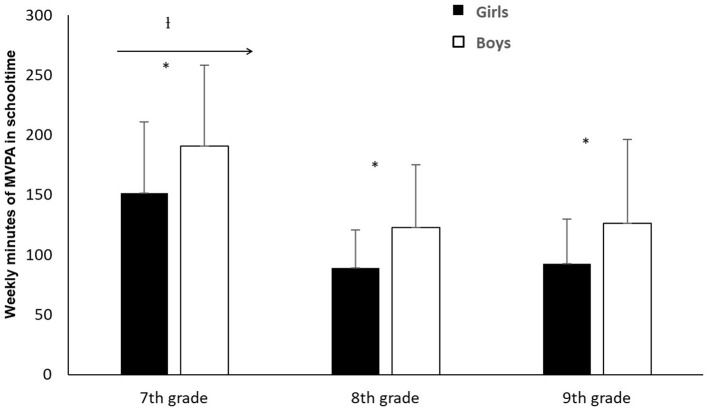
Average weekly MVPA in schooltime for girls/boys + standard deviation.^†^Indicates a significant difference in pupils' MVPA during schooltime between grade 7 and those to the right of the arrow (*p* < 0.001). ^*^Indicates a significant difference between girls' and boys' MVPA in schooltime (*p* < 0.001).

The analyses showed a significant difference in MVPA during schooltime in the period between 7^th^ grade and 9^th^ grade (13–15 years old) (*X*^2^ = 136.68, *p* < 0.001). Follow-up analyses showed a significant difference in MVPA during schooltime from 7^th^ grade to 8^th^ grade (*Z* = −10.80, *p* < 0.001) and from 7^th^ grade to 9^th^ grade (*Z* = −10.19, *p* < 0.001), with MVPA being highest in 7^th^ grade. Further analysis showed that the reduction in MVPA was significant for both genders. In 7^th^ grade, pupils averaged 151.6 (girls) and 190.7 (boys) min in MVPA during schooltime per week. In 8^th^ grade, these numbers dropped to 89.4 for the girls, and 123.2 min for the boys. Further calculation showed this to be a reduction of 41% for the girls and 35.4% for the boys. There was, however, no significant difference in MVPA during schooltime between grades 8 and 9 (*Z* = −0.03, *p* > 0.05). Follow-up analysis showed this applied to both genders. The analyses showed that the boys had significantly more MVPA during school hours than the girls, in 7^th^ grade (*Z* = −4.65, *p* < 0.001), 8^th^ grade (*Z* = −5.33, *p* < 0.001) and 9^th^ grade (*Z* = −4.14, *p* < 0.001). Boys' weekly MVPA in schooltime was 25.8%, 37.8% and 36.1% higher than the girls' in the 7^th^, 8^th^ and 9^th^ grades respectively (Figure 1).

**Figure 2:**
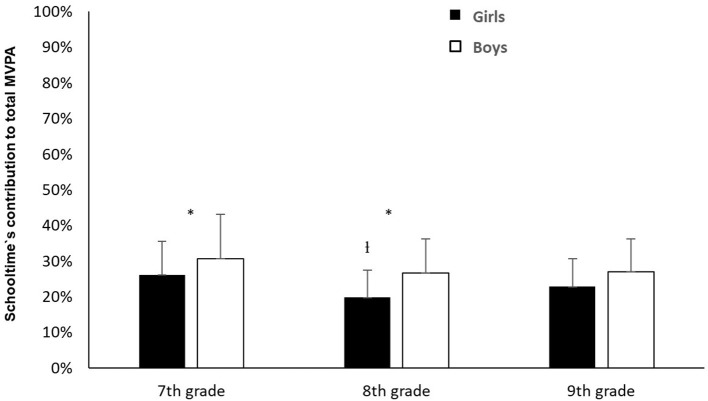
Average percentage of schooltime's weekly contribution of MVPA in relation to the pupils' (girls/boys) total weekly MVPA.^†^Indicates a significant difference in schooltime's contribution to girls' total MVPA between 7^th^ and 8^th^ grade (*p* < 0.001). *Indicates a significant difference in schooltime's contribution of MVPA in relation to total MVPA between girls and boys (*p* < 0.05).

Concerning to the pupils' total MVPA, the analysis showed no significant differences in schooltime's contribution between 7^th^ grade and 9^th^ grade (χ^2^ = 2.31, *p* > 0.05). Nonetheless, the follow-up analysis did show that there were significant differences in schooltime's contribution to the pupils' total MVPA between 7^th^ grade and 8^th^ grade for girls (χ^2^ = 2.31, *p* > 0.05). In 7^th^ grade, schooltime accounted for, respectively, 24.3 and 29.6% of girls' and boys' total MVPA. In 8^th^ grade, this contribution was, respectively, 19.7 and 27.7%, whilst, in 9^th^ grade, it was 22% of the girls' and 25.5% of the boys' total MVPA. The analyses showed that there was a significant gender difference in schooltime's contribution of MVPA in relation to total MVPA, contributing more for the boys in both 7^th^ grade (*Z* = −2.25, *p* < 0.05) and 8^th^ grade (*Z* = −3.55, *p* < 0.001). However, there were no gender differences in schooltime's contribution of MVPA in relation to the pupils' total MVPA in 9^th^ grade (*Z* = −1.87, *p* > 0.05) (Figure 2).

**Figure 3:**
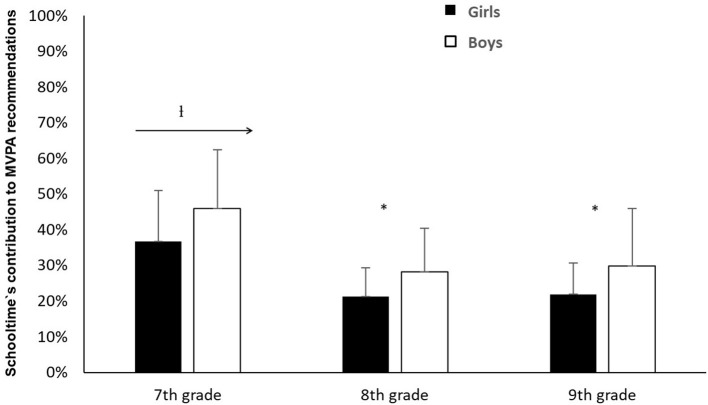
Average percentage of schooltime's weekly contribution of MVPA in relation to the health recommendation of 60 min daily in MVPA for girls/boys + standard deviation.^†^Indicates a significant difference in schooltime's contribution to pupils' MVPA in relation to the health recommendation between 7^th^ grade and those to the right of the arrow between grade 7 and those to the right of the arrow (*p* < 0.001). *Indicates a significant difference in schooltime's contribution to the health recommendation between girls and boys (*p* < 0.001).

The analyses showed a significant difference in schooltime's contribution to the health recommendations of 60 min daily MVPA, in the period between 7^th^ grade and 9^th^ grade (χ^2^ = 139.94, *p* < 0.001). Follow-up analyses showed a significant difference in schooltime's contribution to the health recommendations, between 7^th^ grade and 8^th^ grade (*Z* = −10.83, *p* < 0.001) and from 7^th^ grade to 9^th^ grade (*Z* = −10.20, *p* < 0.001). Further analysis showed that these changes were significant in respect of both genders. On average, weekly schooltime in 7^th^ grade represented 36.1% (girls) and 45.4% (boys) of the MVPA needed to reach the recommended level. In 8^th^ grade, this percentage was down to 21.2% for girls and 29.3% for boys – a percentage decrease of 41.3% and 35.5% from 7^th^ to 8^th^ grade, respectively. There was, however, no significant difference in schooltime's contribution to the health recommendations between grades 8 and 9 (*Z* = −0.04, *p* > 0.05). Follow-up analysis showed that this applied to both genders. The analyses showed that schooltime's contribution to reaching the recommended level was significantly higher for boys than for girls, in 7^th^ grade (*Z* = −4.62, *p* < 0.001), 8^th^ grade (*Z* = −5.33, *p* < 0.001) and in 9^th^ grade (*Z* = −4.13, *p* < 0.001) (Figure 3).

## Discussion

### Changes in the pupils' weekly MVPA during schooltime between 7th and 9th grade

The results showed a significant reduction in weekly MVPA among the pupils between 7^th^ grade and 8^th^ grade, from the age of 13 to the age of 15. This applied to both girls and boys, representing a reduction in PA s during the transition from primary school to secondary school. There were, however, no significant differences in schooltime's contribution to weekly MVPA between 8^th^ grade and 9^th^ grade (13–14 years old). That schooltime's contribution to the pupils' weekly MVPA falls between primary school and secondary school is in agreement with the findings of Remmers et al. (25). Harding et al. (21) also found a significant reduction in pupils' MVPA during schooltime, although their study took only primary school. Compared to the findings in both Remmers' Dutch study and the findings in Hardings' English study, the decline in schooltime's contribution of MVPA, was significantly larger in this study. The smaller reduction in MVPA in Harding et al. (21), can be because the pupils had not moved schools between the two sets of measurements. The difference may also be due to cultural and school policy differences, as suggested by Andersen (19). In his cross-sectional study, Andersen found that schooltime accounted for less MVPA among fifteen-year-olds than among nine-year-olds, and his findings about the fifteen-year-olds MVPA were relatively similar to those in our study. Thus, both our study and the other studies presented here, point to a marked reduction in the contribution to pupils' MVPA made during schooltime after they start secondary school.

Research further suggests that physical education (PE) makes a similar, but quite small contribution to pupils' weekly MVPA in primary and in secondary school (19, 35), with 19 finding that PE lessons accounted for roughly 4%, in both primary and secondary school, of the recommended level of weekly MVPA. As teaching is organized in the same way in both primary and secondary schools (sitting at a desk for 45 min), our findings point in the direction of the changes taking place during recess.

There is evidence that the transition from primary to secondary school is a sensitive period (6). When pupils enter secondary school, there are many new things to deal with, and good activity habits at recess can be one of them. Being physically active may be more difficult because the other pupils are older, it is more difficult to stand out, and where physical play may be seen as childish. An interview study of some pupils from the same sample as the present study found that the transition to secondary school involved getting a new school environment, new teachers, and new classmates to get to know and relate to, as well as increased freedom and new and increased demands (24). The latter findings are in agreement with Ashton (27) which discovered that 7^th^ graders associated secondary school with increased responsibility, freedom, and choices. The increased experience of freedom in secondary school may be related to the possibility to choose where to spend their recess time. While before they were forced to be outside during recess, the pupils can choose to be inside at secondary school. This can allow for greater passivity during recess, as the outdoor area forms both a sanctuary for the pupils and a more conducive environment for physically active play (36). Results from Andersen (19), showed that while 9-year-olds spent 27% of their free time in MVPA, pupils in secondary school spent only 10.5% of their free time in MVPA. Further, the study found that breaks accounted for 18.2 and 6.8%, respectively, of the weekly attainment of the health recommendation for 9-year-old and 15-year-old pupils, respectively. This supports our contention that it is reduced activity during breaks at secondary school that occasions the substantial fall in weekly MVPA during schooltime between primary and secondary school. That our results show no significant difference in MVPA between 8^th^ grade and 9^th^ grade further strengthens this line of argument.

### Schooltime's contribution of weekly MVPA in relation to pupils' total weekly MVPA

The results showed no significant differences, in any of the three sets of measurements, in school time's contribution of weekly MVPA in relation to the total samples' weekly MVPA. However, there were significant differences in schooltime's contribution to weekly MVPA for the girls between 7^th^ and 8^th^ grade, from the age of 13 to the age of 14. The results showed that the contribution of school time weekly MVPA to total weekly MVPA, was under 30% for both genders in 7^th^, 8^th^, and 9^th^ grade. This means that around 70% or more of the pupils' total MVPA occurs in their free time. Considering that MVPA is highest in spring and summer, compared with winter (37), it is worrying that the contribution of weekly MVPA during school time is so low in all three measurements, which were made in spring. It is also concerning that young people's PA behaviour seems to be significantly altered between the final year of primary school and the first of secondary school, especially given that habits formed at a young age are likely to persist into adult (14, 15). The numbers presented in the study by Remmers et al. (25) support this finding, with schooltime contributing 23.5 and 28.5% of, respectively, the girls' and the boys' during their final year in primary school and first year of secondary school. Other research also indicates that the time spent at school contributes a relatively low percentage of the pupils' total MVPA, both in school (38) and in pre-school (16).

Considering that pupils are at school for approximately 40% of their waking time (13), one could expect a larger contribution from schooltime. According to Dale et al. (39) pupils do not compensate for school days with little PA with a higher degree of PA in their free time. In fact, it seems that school days with a higher level of activity contribute to a higher level of activity outside school (39).

### Schooltime's contribution to weekly MVPA attainment of the health authorities' recommended MVPA is concerned?

The results show a significant reduction in schooltime's contribution, toward the recommended level of PA (1, 2), between 7^th^ and 8^th^ grade (from the age of 13 to the age of 14), whilst there was no significant difference between 8^th^ grade and 9^th^ grade. The reduction in schooltime's contribution toward the recommended level of PA from 7^th^ grade to 8^th^ grade, was 41.3% for girls and 35.5% for boys. Results from earlier studies of Norwegian pupils also suggest that the contribution of schooltime to the pupils' attainment of the health recommendation is greater in primary school than in secondary school (19, 20). Longitudinal studies from other countries also support the findings of a reduction through time in school time's contribution to recommended levels of MVPA (21, 25). Thus, the results from this study show that, in all 3 sets of measurements (7^th^, 8^th^, and 9^th^ grade), the school stands for less than half of the number of minutes required to fulfill the recommended weekly amount. Earlier studies have pointed to similar findings (19–21, 25, 26).

### Gender differences in school time MVPA

The results showed that the boys had significantly more MVPA in schooltime than the girls at all three grades, and, further, that the contribution of schooltime to the fulfilment of the recommended level was significantly higher for the boys than for the girls in all three grades. That boys accumulate more MVPA during schooltime is supported by earlier research (19, 20, 25, 26).

Several studies have attempted to clarify the factors contributing to this gender difference in PA. Blatchford et al. (40) found in their study that boys were significantly more involved in ball games during breaks, while girls socialized more with friends in the form of conversations, and as verbal and sedentary play. Their study suggest that the genders use their break time for different activities, with “boys” activities' contributing to more MVPA (40). Girls' and boys' different maturational processes have also been discussed as a possible explanations for these gender differences. The psychological and biological changes occurring in connection with puberty can affect young people's self-perception, which in turn may affect their PA (41). It is possible that these changes may have a negative effect on girls' self-esteem, possibly leading to a reduction of PA. For boys, these changes may lead to an increase in self-esteem, showing itself in increased MVPA (41).

One can argue that such large gender differences in MVPA during schooltime are problematic, especially since the gender differences increase between primary school and secondary school. PE may also help to explain some of the gender differences in schooltime's contribution of MVPA. Several studies indicate that PE is better adapted to boys than to girls (42–44). As the subject is perceived by many as an arena where pupils can engage in the same activities as in organized sports, especially ball activities (45, 46), it is perhaps not unexpected that more boys than girls enjoy PE (46). Because wellbeing and enjoyment are factors in increased PA (47), wellbeing in PE may be thought of as having an impact on differences between boys' and girls' MVPA during school time. This finding gives reason for being particularly attentive to the need for adaptation and differentiation in PE.

The results also show that schooltime's contribution of MVPA in relation to the total weekly MVPA was significantly greater for the boys than for the girls in grades 7 and 8, but not in 9^th^ grade. The boys not only spent more time in MVPA in schooltime in all three measuring periods, but in relation to the pupils' total weekly MVPA, schooltime represented a significantly greater proportion among the boys than the girls. This means that the girls must accumulate a larger proportion of MVPA in their free time. In this way, the school contributes to gender differences.

### Strengths and weaknesses of the study

A clear strength of the study is its use of a longitudinal design, in which reasonably many participants were measured in each of the three school years. All three sets of measurements used standardized and objective measuring instruments, and the test leader, equipment and procedures were the same throughout, and all three measurement periods were at the same time of the year. With longitudinal studies one can expect a certain level of drop out, and a response rate of 56% is therefore satisfactory (48). Statistical analysis (Mann–Whitney *U-*test) of the pupils returning valid results in all three time periods and those who dropped out, showed no significant differences between the two samples (*Z* = −1.57, *p* = 0.12). For this reason, the drop out is seen as being random, and those remaining as being representative of the entire sample. The use of accelerometers as a means of measurement of PA, is seen by many researchers as the best means of measuring PA among children and adolescents (30, 31).

There are, however, certain weaknesses in using accelerometers. They do not tolerate water, which could have significance for the measurement of the pupils' MVPA (30). Another weakness in the use of accelerometers is their limited capacity to register movements involving little vertical acceleration (30). This means that in the case of, for example, cycling or upper-body training, MVPA may be under-recorded. However, there was no possibility of cycling during schooltime in the periods when measurements were being recorded, nor swimming lessons at that time. For practical reasons, a stratified sample of municipalities was used, as there are difficulties with random sampling when conducting studies at this scale. At the same time, there is much that suggests that these two municipalities are not distinctly different from other Norwegian municipalities where gender balance, number of pupils from urban and rural areas, or spread of socioeconomic status are concerned.

## Conclusion

This is one of the very few studies examining schooltimes' contribution of MVPA from a longitudinal perspective, using the same pupils from primary school to secondary school. In this respect, this study presents new knowledge in this area. The results show that the pupils' MVPA during schooltime declines markedly between 7^th^ grade and 8^th^ grade, with a reduction of 41 and 35.4% for the girls and the boys respectively. Our findings also show that schooltime's contribution to the attainment of the weekly recommended level of MVPA, also decrease significantly during the same period. There is, however, no such change between grades 8 and 9, which points to the transition from primary school to secondary school as being vulnerable with respect to the pupils' MVPA. Furthermore, the results show that schooltime contributed more to the boys' MVPA and attainment of the health recommendations than the girls, in all three school years.

As previous research has shown that PA in childhood can establish similar behaviour in adulthood, the school, from a societal and public health perspective, can be an important contributor to public health. Given the findings of this study, practical measures aimed at increasing pupils' MVPA should have a greater focus on secondary schools, especially among girls. The interrelated individual and contextual factors related to the changes in pupils' MVPA in schooltime between 7^th^ grade and 8^th^ grade, should be the subject of further research, and more variables should be included in terms of a better understanding of the PA of children.

## Data availability statement

The raw data supporting the conclusions of this article will be made available by the authors, without undue reservation.

## Ethics statement

The studies involving human participants were reviewed and approved by the Norwegian Centre for Research Data. Written informed consent to participate in this study was provided by the participants' legal guardian/next of kin.

## Author contributions

HM and PL contributed to the design and data collection of the study. PL and MAJ contributed to the analyses. All authors contributed to the introduction, methods, discussion of the study, and approved the submitted version.
